# Childhood obesity trajectories and adolescent mental health: A UK cohort study

**DOI:** 10.1111/ijpo.13206

**Published:** 2025-01-30

**Authors:** I Gusti Ngurah Edi Putra, Michael Daly, Eric Robinson

**Affiliations:** ^1^ Department of Public Health, Policy and Systems, Institute of Population Health University of Liverpool Liverpool UK; ^2^ Department of Psychology Maynooth University Ireland; ^3^ Department of Psychology, Institute of Population Health University of Liverpool Liverpool UK

**Keywords:** mental health, obesity history, obesity trajectory, psychological well‐being, weight change

## Abstract

**Background:**

There is limited evidence on how changes in obesity from childhood to adolescence are associated with adolescent mental health. We examined the associations between childhood obesity trajectories, obesity episodes, and mental health at age 17.

**Methods:**

Data were from the UK Millennium Cohort Study. Obesity trajectory groups at ages 7 and 17 (*n* = 8306) and previous obesity episodes (number of sweeps with obesity) at ages 7, 11 and 14 (*n* = 7246) were examined. Caregiver and self‐reported internalising and externalising symptoms at age 17 were used to measure mental health. Linear regression models were used.

**Results:**

Relative to never developing obesity, obesity development (*β* = 1.01; 95% CI = 0.71, 1.32) and persistence (*β* = 1.18; 95% CI = 0.74, 1.61) were associated with higher internalising symptoms at age 17 and worsening (increase in scores) of these symptoms between ages 7 and 17 (*β* = 0.87; 95% CI = 0.57, 1.17 and *β* = 0.86; 95% CI = 0.56, 1.26 for development and persistence, respectively). Obesity development was associated with higher externalising symptoms at age 17 (*β* = 0.52; 95% CI = 0.25, 0.80) and worsening of these symptoms over time (*β* = 0.30; 95% CI = 0.07, 0.53). Having multiple past obesity episodes was not associated with worsening mental health independent of follow‐up weight status. There were no differences in mental health outcomes between children who reversed versus never developed obesity.

**Conclusions:**

Obesity development or persistence from ages 7 to 17 are associated with worsening mental health. If childhood obesity is reversed, there appears to be no evidence of a negative association between previous obesity and mental health at age 17.

## 1 INTRODUCTION

Childhood obesity and its associated disease burden are now widely considered to be an urgent worldwide epidemic.^1^ As well as contributing to physical health outcomes, childhood obesity has a significant mental health burden. Numerous studies show that children with obesity are more likely than others to suffer from worse mental health,^2^, ^3^ and a smaller subset of studies suggest that childhood obesity is a prospective risk factor for declines in mental health.^4^, ^5^, ^6^

Although studies suggest childhood obesity tracks into adolescence and adulthood,^7^ the extent to which the mental health burden associated with obesity in childhood may continue into later life is unclear. Living with obesity during childhood could be particularly damaging to long‐term psychological well‐being and create negative and persistent personal attributions relating to body image and self‐esteem.^6^, ^8^, ^9^ In line with this, a study of older Icelandic adults found that having overweight or obesity in childhood was associated with an increased risk of lifetime major depressive disorder.^10^ Similarly, amongst US adults, an earlier history of obesity in adulthood was shown to be predictive of worse mental health.^11^ Although these findings are consistent with the possibility that earlier life obesity could have long‐term implications for mental health, a recent systematic review concluded that there is a lack of methodologically appropriate studies addressing this question.^12^ This includes a limited number of studies controlling for baseline childhood mental health and how different trajectories of childhood obesity are associated with adolescent mental health.^12^

The extent to which childhood obesity may affect longer‐term mental health is likely to be in part dependent on the trajectory of obesity during childhood,^13^, ^14^ but there is a lack of formal testing of this proposition. Some children develop obesity prior to adolescence (e.g., before age 11) and remain within the obesity weight status category into adulthood. This trajectory may result in repeated long‐term exposure to obesity stigma or weight‐based teasing,^8^, ^15^ and therefore be particularly damaging to longer‐term mental health. Conversely, other children with obesity may lose weight during their teenage years and by the end of adolescence (e.g., age 17) no longer live with obesity. The extent to which reversal of obesity would fully attenuate any previous damaging psychological effects of living with obesity or still be associated with poorer mental health is unclear. There is also limited evidence of whether living with childhood obesity over a longer period (e.g., over multiple years) is associated with worsening long‐term mental health independently of current obesity weight status. Another important trajectory is the development of obesity during the teenage years, which may confer separate increased risk for mental health due to teenage years being a period of life in which social acceptance and body image concerns are high.^16^, ^17^

To date, there has been limited prospective research examining how changes in obesity status or obesity trajectories relate to long‐term mental health and the extent to which having past obesity episodes is associated with long‐term mental health independently of current weight status. In the present research, we made use of nationally representative birth cohort data of UK children and examined how obesity trajectories between ages 7 and 17 relate to measures of mental health from ages 7 to 17. Furthermore, to more directly test the question of whether obesity during childhood has longer‐term associations with mental health, we examined the extent to which previous episodes of living with obesity during childhood (ages 7–14) were predictive of mental health at age 17.

## 2 METHODS

### 2.1 Study design and sample

We used data from the Millennium Cohort Study (MCS), a nationally representative birth cohort study of UK children born in 2000–2002. Children (*n* = 18 827) and their families were recruited from the Child Benefit register using stratified cluster sample design, taking into account stratifications by countries (England, Wales, Scotland, Northern Ireland), disadvantaged communities, and ethnicity (in England) (see References [18, 19, 20] for more detailed information on MCS). Data collection has been conducted over seven sweeps, corresponding to ages 9 months, 3, 5, 7, 11, 14 and 17 years. Data collection for the most recent sweep at age 23 years (Sweep 8) is currently ongoing. Based on previous studies,^4^, ^5^ including one study using MCS data,^4^ the comorbidity between unhealthy weight and mental health problems occurs from 7 years old onwards. Therefore, our study included data of children from Sweep 4 (7 years). Only the first child or first cohort from recruited families was included in the analyses to avoid clustering by family,^21^, ^22^ excluding a small number of twins and triplets (*n* = 166 or around 1% from the total number of recruited children). MCS' data collection procedure and contents received ethics approval from the National Health Service Research Ethics Committee and the National Research Ethics Service. Informed consent was obtained from caregivers when children were younger than 17 years and directly from the cohort members at age 17.

First, we examined whether obesity trajectories were linked to changes in mental health. To define these trajectories, we used data on obesity status (yes, no) from two sweeps (baseline and follow‐up). Based on changes in obesity status, the trajectories were categorised as never developing obesity (no obesity in either sweep), developing obesity (no obesity at baseline but obesity at follow‐up), reversing obesity (obesity at baseline but not at follow‐up) and continuous obesity (obesity in both sweeps). We explored inclusion of interim sweeps in these analyses, but patterns of trajectory were similar when using two sweeps. We examined obesity trajectories using data at ages 7 and 17. For this analysis, we included participants whose weight status was available in both ages 7 and 17, resulting in a maximum analytical sample size of 8306.

Second, because we were concerned that trajectories based solely on baseline and follow‐up data may fail to account for duration of obesity in children, we investigated whether having multiple previous obesity episodes (i.e., the number of sweeps during which children were observed to have obesity) may be important for long‐term mental health. We used data from three sweeps at ages 7, 11 and 14 to classify previous obesity episodes into three categories: never developing obesity, obesity at one time point and obesity at multiple (two and three) time points. We then examined the association between these previous obesity episodes and mental health at age 17. For this second analysis, we only included the cohort members whose weight status was consistently available at ages 7, 11, 14 and 17, with a maximum analytical sample size of 7246.

### 2.2 Obesity status

Weight and height (in light clothing without shoes) of cohort members assessed by trained interviewers using standardised procedures were used to calculate body mass index (BMI) in kg/m^2^.^23^ We excluded participants with outlier BMI (<10 or >50).^24^ BMI at each sweep was standardised into BMI *z*‐scores taking into account sex and age at the time of assessment,^25^ following the British 1990 child growth reference population (UK90) which is more appropriate for the UK context.^26^ Obesity status was then defined using the UK90 reference percentile or centile cut‐offs (2nd centile or below for underweight, between above 2nd centile and below 85th centile for normal weight, between 85th centile and below 95th centile for overweight and 95th centile or above for obesity).^24^, ^27^, ^28^ As we aimed to examine the association between obesity trajectories and mental health, and underweight is associated with poorer psychological well‐being,^29^ we omitted cohort members with underweight (1 to 2%) from the analyses.

### 2.3 Mental health

Four deficit‐focused domains (emotional symptoms, peer problems, conduct problems, hyperactivity) from the Strengths and Difficulties Questionnaire (SDQ)^30^ reported by caregivers at ages 7 and 17 were used to assess internalising and externalising symptoms. The SDQ is a validated tool which has been widely used to screen mental health in diverse settings.^31^, ^32^, ^33^ Each of the four deficit‐focused domains consists of five 3‐point Likert‐scale items (e.g., “Many worries, often seems worried”, “Often fights with other children or bullies them”) (responses were scored as 0 = “not true”, 1 = “somewhat true” and 2 = “certainly true”) with a total score ranging from 0 to 10. Internalising symptoms were defined by adding together emotional and peer problems, and the remaining problem domains, conduct problems and hyperactivity, constituted externalising symptoms. Therefore, each symptom has a total score from 0 to 20 with a higher score indicating worse mental health problems.^24^

### 2.4 Covariates

For the first analysis on obesity trajectories and mental health using data at ages 7 and 17, baseline (age 7) sociodemographic covariates were controlled: sex (male; female), race (White; non‐White), family type (one‐; two‐caregiver family), caregiver education (classified into five National Vocational Qualification—NVQ scales (see^34^): NVQ level 1: “Certificate of Secondary Education—CSE below grade 1; General Certificate of Secondary Education—GCSE or O Level below grade C; The Scottish Certificate of Education—SCE Standard, Ordinary grades below grade 3 or Junior Certificate below grade C”, NVQ level 2: “GCSE or O Level grade A‐C; SCE Standard, Ordinary grades 1–3 or Junior Certificate grade A–C”, NVQ level 3: “A/AS/S Levels/SCE Higher; Scottish Certificate Sixth Year Studies; Leaving Certificate”, NVQ level 4: “teaching qualification below degree level to first‐degree qualification”, NVQ level 5: “higher degree and postgraduate qualification”, and additional groups for no qualification and overseas qualification),^22^, ^24^ occupation (grouped into managerial and professional; intermediate; small employers and self‐employed; lower supervisory and technical; semi‐routine and routine occupations, and unemployed),^24^, ^34^ family income (quintiles of equivalised income)^8^ and baseline BMI *z*‐scores. We used derived variables created by the data custodian to gauge caregiver education, occupation, and family income.^34^ The second analysis assessed the role of previous obesity episodes from ages 7 to 14 (three sweeps) in predicting current mental health at age 17, and we controlled for covariates age 17, except for caregiver education, occupation, and income available at age 14. In all the analyses, we also controlled for pubertal status reported at age 11, defined as menstruation in females and voice deepening in males.^24^

### 2.5 Data analysis

Overall longitudinal sample weights were available and created by the data custodian across sweeps.^20^ At Sweep 1, longitudinal weight was the same as the sample (design) which was created to account for unequal probabilities of selection due to stratified cluster sample design.^20^ For Sweep 2 and thereafter, the longitudinal weight was produced by multiplying the longitudinal weight at the previous sweep with a non‐response weight (i.e., the inverse probability of responding) for the current weight.^20^ Following guidelines,^20^ we present weighted percentages (%), mean, and standard deviation (SD) for participants' sociodemographic characteristics. For longitudinal analysis, where the analytical sample size decreases due to increasing missing values over time and the exclusion of baseline data without follow‐up, we applied the longitudinal sample weights from the last sweep (follow‐up at age 17). These weights have been adjusted for sample attritions from previous sweeps (ages 7 to 14) and non‐response at age 17 (follow‐up).^20^ Using the longitudinal sample weights from age 17 will provide more representative estimates. This approach of using the last sweep's longitudinal sample weights has been recommended for analysis with multiple data collection sweeps.^35^ These sample weights were further incorporated into the multiple imputation process to address missing data more effectively.

We used multiple imputations by chained equation (MICE)^36^, ^37^ to fill in missing values in covariates and mental health outcomes (up to 17% from the maximum analytical sample size). Missing at random was assumed as some variables in the dataset, including household income and family structure, predicted missingness. We used STATA to create 20 inputted datasets. In addition to including the main variables, our imputation model also fitted some auxiliary variables, including cohort members' and caregivers' general health and longstanding illness status, caregivers' perception of financial difficulty, number of people living in the house, housing tenure, and disadvantage stratification (i.e., stratum; the countries—England, Wales, Scotland, Northern Ireland—were classified as either disadvantaged or advantaged)^22^, ^24^ to improve the imputation model. Following published guidelines,^38^, ^39^ the imputation model also included non‐response‐adjusted longitudinal sample weights at the last sweep (age 17). Imputed datasets were then set up for complex survey design analysis to account for clustering and stratification.^38^

Linear regression models were used to assess the association between obesity trajectories (obesity changes at two‐time points: ages 7 and 17; presented as never developing obesity, developing obesity, reversing obesity, and continuous obesity) and mental health outcomes (internalising and externalising symptoms) at age 17, controlling for covariates. Additional models were created to examine longitudinal changes in mental health by controlling for baseline mental health at age 7. We also examined whether reversing obesity may benefit mental health by comparing mental health outcomes between those who reversed obesity versus remained living with obesity from age 7 to 17.

We also used linear regression to examine the association between the number of previous obesity episodes at ages 7, 11 and 14 and mental health at age 17, controlling for covariates. Mental health at age 7 was controlled in additional models to assess changes in mental health outcomes. We stratified the analysis by whether cohort members were living with versus without obesity at age 17 to assess the role of past obesity episodes on current mental health whilst accounting for current obesity status. Findings from regression analyses were reported as regression coefficient (*β*), 95% confidence intervals, and *p*‐value. Findings with *p*‐value <0.05 were considered statistically significant.

We conducted additional sensitivity analyses. We tested whether findings for obesity trajectories and previous obesity episodes remained consistent when mental health at age 17 was self‐reported by the cohort members (as opposed to caregiver‐reported mental health at age 17 as the outcome). Self‐reported mental health (SDQ) was only available at age 17. For the first analysis on obesity trajectories, a small number of participants remained living with obesity (*n* = 663) or reversed obesity (*n* = 312) at age 17 because of a relatively low prevalence of obesity at age 7 as baseline (see Table 1). Therefore, we conducted a sensitivity analysis using age 11 as baseline (as opposed to age 7) to increase analytic sample sizes (*n* = 1088 and 537 for continuous and reversing obesity, respectively). Compared to only 13% of the cohort members living with obesity at age 7, the prevalence of obesity increased to 20% at age 11 and then stabilised at age 14.^28^ For the second analysis on previous obesity episodes, we conducted a sensitivity analysis to examine whether the association between past obesity episodes and current mental health attenuated after controlling for current obesity status at age 17 using a full analytical sample size (as opposed to stratified by obesity status at age 17).

**TABLE 1:** Baseline characteristics of participants (*n* = 8306).

Variables	*n*	Unweighted %	Weighted %^a^
Sociodemographic characteristics			
Sex	8306		
Male	4075	49.06	49.43
Female	4231	50.94	50.57
Race	8305		
White	6869	82.71	84.53
Non‐White	1436	17.29	15.47
Family structure	8306		
One‐caregiver family	1397	16.82	17.80
Two‐caregiver family	6909	83.18	82.20
Caregiver education	8306		
No qualification	487	5.86	5.89
Overseas qualification	166	2.00	1.91
NVQ level 1	301	3.62	3.84
NVQ level 2	1685	20.29	21.43
NVQ level 3	1289	15.52	15.34
NVQ level 4	3292	39.63	39.19
NVQ level 5	1086	13.07	12.39
Caregiver occupation	8306		
Unemployed	228	3.47	3.56
Semi‐routine and routine	1540	18.54	18.31
Low supervisory, technical	548	6.60	6.74
Small employers, self‐employed	662	7.97	8.24
Intermediate	1086	13.07	12.93
Managerial, professional	4182	50.35	50.22
Family income	8229		
Lowest	1445	17.41	16.64
Second	1554	18.73	17.73
Third	1648	19.86	19.49
Fourth	1797	21.65	22.23
Highest	1855	22.35	23.91
Obesity status			
Obesity trajectories using data at ages 7 and 17	8306		
Never developed obesity	6201	74.66	75.95
Developed obesity	1130	13.60	13.56
Reversed obesity	312	3.76	3.38
Continuous obesity	663	7.98	7.11
Past obesity episodes using data at ages 7, 11 and 14^b^	7246		
Never developed obesity	5408	74.63	76.18
Obesity at one time	738	10.18	9.62
Obesity at two or three times	1100	15.18	14.20
Mental health at age 7			
Internalising symptoms (a possible range of 0–20)	8088		
Mean (SD)		2.60 (2.68)	2.62 (2.66)
Externalising symptoms (a possible range of 0–20)	8075		
Mean (SD)		4.43 (3.47)	4.52 (3.53)
Mental health at age 17			
Internalising symptoms (a possible range of 0–20)	7544		
Mean (SD)		3.64 (3.40)	3.62 (3.44)
Externalising symptoms (a possible range of 0–20)	7542		
Mean (SD)		3.49 (3.21)	3.46 (3.20)

Abbreviations: %, percentages; *n*, sample size; NVQ, National Vocational Qualification; SD, standard deviation.

^a^ Weighted values were calculated incorporating longitudinal sample weights (see Section 2.5).

^b^ Maximum analytical sample size was 7246 (see Section 2.1).

## 3 RESULTS

There were equivalent proportions of males and females (Table 1). Most of the participants were of White race and resided with two caregivers. 10% of participants had obesity at one time point and 14% experienced two or three time points of obesity between ages 7 and 14. Internalising symptoms increased between ages 7 and 17, whilst externalising symptoms declined.

Table 2 presents the associations between obesity trajectory groups and mental health at age 17 (Model 1) and changes in mental health between ages 7 and 17 (Model 2). Relative to never developing obesity, cohort members who had developed obesity (*β* = 1.01; 95% CI = 0.71, 1.32) and remained living with obesity (*β* = 1.18; 95% CI = 0.74, 1.61) had greater internalising symptoms at age 17. Similarly, these trajectory groups also experienced an increase in internalising symptoms between ages 7 and 17 (*β* = 0.87; 95% CI = 0.57, 1.17 and *β* = 0.86; 95% CI = 0.56, 1.26 for developing obesity and continuous obesity trajectories, respectively). However, internalising symptoms were not different between cohort members who no longer had obesity at age 17 versus their counterparts who never developed obesity (*β* = 0.24; 95% CI = −0.24, 0.73), even after controlling baseline internalising symptoms (*β* = 0.07; 95% CI = −0.38, 0.53). This indicates that reversing obesity may have benefits for improving mental health. Findings were largely similar but with smaller effect sizes when self‐reported mental health at age 17 (as opposed to caregiver‐reported mental health) was examined as the outcome (Table S1). The findings are supported by our analysis limited to participants with obesity at age 7 (Table 3). We found that obesity reversal at age 17 (vs. remaining with obesity) was associated with lower age‐17 (*β* = −0.83; 95% CI = −1.39, −0.28) and decreased age‐7‐to‐17 internalising symptoms (*β* = −0.78; 95% CI = −1.26, −0.28) (Table 3). Examining self‐reported mental health at age 17 also produced consistent evidence (Table S2). Analyses using age 11 as a baseline showed similar findings for internalising symptoms (see Tables S3 and S4).

**TABLE 2:** Associations between trajectory groups of obesity and mental health at age 17 (*n* = 8306).

Obesity trajectories (baseline at age 7 and follow‐up at age 17) (ref = never developed obesity)	Model 1			Model 2		
	*β*	95% CI	*p*‐value	*β*	95% CI	*p*‐value
Dependent variable: internalising symptoms						
Developed obesity	1.01	0.71, 1.32	<0.001	0.87	0.57, 1.17	<0.001
Reversed obesity	0.24	−0.24, 0.73	0.325	0.07	−0.38, 0.53	0.758
Continuous obesity	1.18	0.74, 1.61	<0.001	0.86	0.56, 1.26	<0.001
Dependent variable: externalising symptoms						
Developed obesity	0.52	0.25, 0.80	<0.001	0.30	0.07, 0.53	0.009
Reversed obesity	−0.10	−0.58, 0.38	0.679	0.13	−0.29, 0.56	0.545
Continuous obesity	0.24	−0.17, 0.65	0.255	0.13	−0.23, 0.49	0.483

*Note*: Internalising and externalising symptoms ranged from 0 to 20. Separate regression models were developed for each mental health outcome. Model 1 controlled for baseline sex, race, family structure, caregiver education, caregiver employment, family income, pubertal status at age 11, and baseline BMI *z*‐score at age 7. Model 2: Model 1 with an additional adjustment for baseline mental health at age 7 to assess changes in mental health from age 7 to 17.

Abbreviations: *β*, regression coefficient; CI, confidence intervals; ref, reference group.

**TABLE 3:** Associations between reversed (vs. continuous) obesity and mental health at age 17 (*n* = 975).

Obesity trajectories (baseline at age 7 and follow‐up at age 17) (ref = continuous obesity)	Model 1			Model 2		
	*β*	95% CI	*p*‐value	*β*	95% CI	*p*‐value
Dependent variable: internalising symptoms						
Reversed obesity	−0.83	−1.39, −0.28	0.003	−0.78	−1.26, −0.28	0.002
Dependent variable: externalising symptoms						
Reversed obesity	−0.38	−0.85, 0.08	0.108	−0.05	−0.48, 0.39	0.835

*Note*: Internalising and externalising symptoms ranged from 0 to 20. Separate regression models were developed for each mental health outcome. Model 1 controlled for baseline sex, race, family structure, caregiver education, caregiver employment, family income, pubertal status at age 11, and baseline BMI *z*‐score at age 7. Model 2: Model 1 with an additional adjustment for baseline mental health at age 7 to assess changes in mental health from age 7 to 17.

Abbreviations: *β*, regression coefficient; CI, confidence intervals; ref, reference group.

Children without obesity at age 7 who had developed obesity at age 17 (relative to never developing obesity) had greater (*β* = 0.52; 95% CI = 0.25, 0.80) and increased externalising symptoms (*β* = 0.30; 95% CI = 0.07, 0.53) (Table 2). However, no differences in externalising symptoms for those who reversed or continued living with obesity by age 17 versus never developing obesity were observed. We found consistent evidence that developing obesity was associated with higher externalising symptoms at age 17, but not statistically significant for increasing these symptoms when self‐reported mental health was examined (Table S1). Children who transitioned into non‐obesity weight status and remained living with obesity from ages 7 to 17 had no differences in follow‐up (*β* = −0.38; 95% CI = −0.85, 0.08) and changes in externalising symptoms (*β* = −0.05; 95% CI = −0.48, 0.39) (Table 3). However, findings from examining self‐reported mental health showed that reversing obesity was statistically significantly associated with lower externalising symptoms at age 17 (*β* = −0.74; 95% CI = −1.31, −0.17) (Table S2). Findings from additional analysis examining obesity trajectories using age 11 as baseline showed that developing obesity and remaining living with obesity were statistically significantly associated with higher externalising symptoms at age 17, but not associated with changes in externalising symptoms (Table S3). No differences in externalising symptoms between those who reversed obesity and remained living with obesity from ages 11 to 17 were observed (Table S4).

We tested whether the number of past obesity episodes from ages 7 to 14 was associated with current mental health at age 17 in participants with and without current obesity (Table 4). We found no evidence that having multiple previous obesity episodes from ages 7 to 14 was associated with worse internalising and externalising symptoms by age 17 or changes in both symptoms from ages 7 to 17 in participants with and without current obesity. Findings were similar for self‐reported mental health (Table S5). The lack of evidence of the association between past obesity episodes from ages 7 to 14 and current mental health at age 17 in cohort members with current obesity or without current obesity may be due to the more important role current obesity status has on current mental health. We conducted a sensitivity analysis using a full analytical sample size to examine whether past obesity episodes are associated with current mental health after controlling for current obesity status. Although having two or more past episodes of obesity (compared to never developing obesity) was associated with higher internalising symptoms at age 17 and an increase in these symptoms between ages 7 and 17, these associations became null after adjusting for current obesity status at age 17 (Table S6).

**TABLE 4:** Associations between the number of previous obesity episodes from ages 7 to 14 and current psychological measures in participants with and without current obesity at age 17.

Previous obesity episodes from ages 7 to 14 (ref = never developed obesity)	Participants with obesity at age 17 (*n* = 1479)						Participants without obesity at age 17 (*n* = 5767)					
	Model 1			Model 2			Model 1			Model 2		
	β	95% CI	p‐value	β	95% CI	p‐value	β	95% CI	p‐value	β	95% CI	p‐value
Dependent variable: internalising symptoms												
Obesity at one time	−0.25	−0.89, 0.39	0.444	−0.56	−1.15, 0.04	0.068	0.18	−0.20, 0.55	0.356	0.03	−0.33, 0.38	0.883
Obesity at two/three times	−0.12	−0.65, 0.40	0.639	−0.31	−0.82, 0.21	0.246	−0.02	−0.52, 0.48	0.943	−0.05	−0.49, 0.39	0.824
Dependent variable: externalising symptoms												
Obesity at one time	−0.14	−0.73, 0.45	0.640	−0.25	−0.79, 0.29	0.359	0.09	−0.31, 0.49	0.674	0.05	−0.29, 0.39	0.788
Obesity at two/three times	−0.45	−0.99, 0.08	0.096	−0.43	−0.89, 0.04	0.074	0.01	−0.43, 0.45	0.956	0.04	−0.35, 0.42	0.857

*Note*: Internalising and externalising symptoms ranged from 0 to 20. Separate regression models were developed for each mental health outcome. Model 1 controlled for current sex, race, family structure, caregiver education, caregiver employment, family income, and puberty status at age 11. Model 2: Model 1 with an additional adjustment for mental health at age 7 to assess changes in mental health from age 7 to 17.

Abbreviations: *β*, regression coefficient; CI, confidence intervals; ref, reference group.

## 4 DISCUSSION

We examined associations between obesity weight status trajectories, past obesity episodes, and mental health in UK children between ages 7 and 17. Compared to children without obesity at both time points studied, children who had developed obesity at age 17, exhibited higher internalising and externalising symptoms at age 17 and worsening of both symptoms between ages 7 and 17. Children who remained living with obesity versus never developed obesity had higher internalising symptoms (but not externalising symptoms) at age 17 and worsening internalising symptoms between ages 7 and 17. Children with obesity at age 7 whose obesity was subsequently reversed at age 17 did not have significantly different internalising and externalising symptoms at age 17 compared to children who never developed obesity. Findings were largely consistent whether caregiver or self‐reported mental health was examined, and when obesity and mental health trajectories were assessed from age 11 (as opposed to 7).

Consistent with our primary analysis, we found that the number of past episodes of obesity during ages 7–14 was not significantly associated with mental health (internalising and externalising symptoms) in either children with or without obesity at age 17, indicating obesity status at age 17 may have a more important role for mental health than previous experiences. Likewise, there was no evidence of past episodes of obesity being linked to mental health at age 17, independent of current obesity status at age 17. Collectively, these findings suggest that the extent to which childhood obesity is associated with a longer‐term risk of worse adolescent mental health may be largely dependent on the extent to which a child's obesity persists over time or develops in adolescence. By age 17 at least, experiencing obesity during childhood does not appear to be associated with a long‐term risk of worse mental health independent of current weight status in the present study. No evidence of a negative association of previous obesity on mental health at age 17 was apparent if childhood obesity had been reversed.

The present findings are consistent with previous research suggesting that both the development and persistence of obesity are associated with worse mental health.^14^ A relatively small number of studies have suggested that obesity during childhood and adolescent may confer long‐term risk for worse mental health into adulthood.^10^, ^40^ However, studies have tended to be limited in methodological quality and this has made temporal and causal inference difficult.^12^ Unlike a limitation of many previous studies, a strength of the present work was that both objectively measured weight status and validated reports of mental health symptoms were available at every time point examined. This approach allowed us to account for both previous mental health symptoms and obesity status when examining mental health at age 17. Findings are consistent with obesity during childhood being a risk factor for later worse mental health, but primarily when obesity has tracked from (or developed during) childhood into early adulthood (age 17). This interpretation is consistent with the notion that early intervention in childhood obesity may benefit both physical and mental health and highlights the need for childhood obesity prevention and treatment policies.

There may be a range of psychosocial factors that explain why living with obesity is predictive of worsening mental health, including but not limited to the effects that the stigma of obesity may have on self‐esteem and body image.^3^, ^9^ Similarly, children with obesity experience weight‐related teasing and bullying, which are likely detrimental to mental health.^41^ In addition, parental identification of child overweight and obesity has also been shown to be associated with worse child mental health.^42^ This highlights the need for parents to be made aware of the psychological burden associated with childhood obesity and adequate mental health support for families and children living with obesity.

In this study, associations between obesity development, continuation (vs. never developing obesity) and worsening mental health were stronger for internalising than externalising symptoms. Similarly, unhealthy weight and internalising symptoms have been found to codevelop with each other in childhood and adolescence.^4^, ^5^ Stronger associations for internalising than externalising symptoms may be explained by subsequent consequences of living with obesity that involve more negative self‐perception and internal thought processes. Children living with obesity, often experience peer bullying and weight stigma that may cause low self‐esteem and body dissatisfaction, which can be key contributors to elevated emotional or internalising problem behaviours (e.g., anxiety, depression).^6^, ^8^, ^43^, ^44^ As living with obesity may be also associated with withdrawal from social environments,^45^ this may limit externalising behaviours that often involve more outward‐facing interactions. On the other hand, experiencing weight loss (e.g., reversing obesity trajectory) may improve body satisfaction and in doing so reduce emotional distress.^46^ More research is warranted to better understand potential underlying mechanisms of the present findings.

We found that previous obesity episodes may not be associated with long‐term mental health independently of current weight status. It would be preferable in future research to examine the relationship between childhood obesity trajectories and mental health beyond age 17. For example, it is feasible that persistence of obesity over long time periods (e.g., during childhood and adulthood) could be psychologically ‘scarring’ to mental health, independent of current obesity status,^11^ as adults who have lost weight and reversed their obesity report may still feel stigmatised.^47^

Strengths of the research include the use of nationally population‐based cohort data and repeat objective measurements of body weight. We also conducted a sensitivity analysis using self‐reported mental health and found results were largely consistent. However, reversal of obesity tended to be associated with improvements in externalising and internalising symptoms when data was analysed using this approach and further research to explain differences in findings between parent versus child reported mental health analyses is now warranted. A limitation of the present research was that although we examined both internalising and externalising symptoms and these are strongly predictive of a range of mental health and psychiatric disorder conditions, data on formal (e.g., physician) diagnosis of a mental health condition(s) were not available. BMI z‐scores are widely used to identify childhood obesity, but do not account for body composition.^48^ Inclusion of other measurements, such as body fat would provide a better characterisation of level of adiposity.^49^ In addition, we defined pubertal status at age 11 as menstruation in female and voice deepening in males.^24^ This may not accurately capture pubertal status in all children, particularly in females who start their puberty onset 2 years before experiencing menstruation.^50^ Finally, because the sample was predominantly White, findings may therefore not to generalise to other cultural contexts and ethnicities.

## 5 CONCLUSIONS

Both the development and persistence of obesity from ages 7 to 17 are associated with worsening mental health. However, if childhood obesity is reversed there appears to be no evidence of a negative association between previous obesity and mental health at age 17.

## AUTHOR CONTRIBUTIONS

IGNEP, MD, and ER conceptualised the study. IGNEP carried out the initial analysis. IGNEP and ER drafted the initial manuscript. MD and ER critically reviewed and revised the manuscript. All authors approved the final manuscript as submitted.

## FUNDING INFORMATION

This work was supported by the Economic and Social Research Council (ESRC) (ES/V017594/1). The funder had no role in the study design, analysis, manuscript preparation, or decision to publish.

## CONFLICTS OF INTEREST

The other authors have no conflicts of interest to disclose.

## Supporting information


**TABLE S1:** Associations between trajectory groups of obesity and self‐reported follow‐up mental health at age 17 (*n* = 8306) (see Table 2 in the main document for examining caregiver‐reported follow‐up mental health).
**TABLE S2.** Associations between reversing (vs. remaining living with) obesity and self‐reported follow‐up mental health at age 17 (*n* = 975) (see Table 3 in the main document for examining caregiver‐reported follow‐up mental health).
**TABLE S3.** Associations between trajectory groups of obesity and mental health between ages 11 and 17 (*n* = 8469).
**TABLE S4.** Associations between reversing (vs. remaining living with) obesity and mental health between ages 11 and 17 (*n* = 1625).
**TABLE S5.** Associations between the number of previous obesity episodes from ages 7 to 14 and current self‐reported follow‐up mental health in participant with and without current obesity at age 17 (see Table 4 in the main document for examining caregiver‐reported follow‐up mental health).
**TABLE S6.** Associations between the number of previous obesity episodes from ages 7 to 14 and current mental health at age 17 (*n* = 7246).

## Data Availability

Data are available at https://doi.org/10.5255/UKDA-Series-2000031 with the permission of UK Data Service.
