# Supplementary material for: Childhood obesity trajectories and adolescent mental health: A UK cohort study
Source: Pediatr Obes. 2025 Jan 30;20(3):e13206. doi: 10.1111/ijpo.13206 (PMC11803180; doi:10.1111/ijpo.13206)
Supplement: Supplementary file 1 — TABLE S1: Associations between trajectory groups of obesity and self‐reported follow‐up mental health at age 17 (n = 8306) (see Table 2 in the main document for examining caregiver‐reported follow‐up mental health). TABLE S2. Associations between reversing (vs. remaining living with) obesity and self‐reported follow‐up mental health at age 17 (n = 975) (see Table 3 in the main document for examining caregiver‐reported follow‐up mental health). TABLE S3. Associations between trajectory groups of obesity and mental health between ages 11 and 17 (n = 8469). TABLE S4. Associations between reversing (vs. remaining living with) obesity and mental health between ages 11 and 17 (n = 1625). TABLE S5. Associations between the number of previous obesity episodes from ages 7 to 14 and current self‐reported follow‐up mental health in participant with and without current obesity at age 17 (see Table 4 in the main document for examining caregiver‐reported follow‐up mental health). TABLE S6. Associations between the number of previous obesity episodes from ages 7 to 14 and current mental health at age 17 (n = 7246). [file IJPO-20-e13206-s001.pdf]

Supplementary information

Childhood obesity trajectories and adolescent mental health: A UK cohort study

I Gusti Ngurah Edi Putra[1], Michael Daly[2], Eric Robinson[3]

[1]Department of Public Health, Policy and Systems, Institute of Population Health, University of Liverpool, Liverpool, UK

[2]Department of Psychology, Maynooth University, Maynooth, Ireland

[3]Department of Psychology, Institute of Population Health, University of Liverpool, Liverpool, UK

Correspondence

I Gusti Ngurah Edi Putra, Department of Public Health, Policy and Systems, Institute of Population Health, Whelan Building, Quadrangle, The University of Liverpool, Liverpool, L69 3GB, i.gusti.ngurah.edi.putra@liverpool.ac.uk

Table S1. Associations between trajectory groups of obesity and self-reported follow-up mental health at age 17 (n= 8,306) *(see Table 2 in the main document for examining caregiver-reported follow-up mental health)*

**Obesity trajectories (baseline at age 7 and follow-up at age 17)** ***(ref = never developed obesity)***	**Model 1**			**Model 2**		
	**β**	**95% CI**	**p-value**	**β**	**95% CI**	**p-value**
**Dependent variable: Internalising symptoms**						
Developed obesity	0.62	0.34, 0.91	<0.001	0.57	0.29, 0.83	<0.001
Reversed obesity	-0.10	-0.61, 0.41	0.705	-0.18	-0.68, 0.32	0.483
Continuous obesity	0.91	0.44, 1.38	<0.001	0.77	0.31, 1.22	0.001
**Dependent variable: Externalising symptoms**						
Developed obesity	0.35	0.07, 0.62	0.013	0.23	-0.03, 0.49	0.089
Reversed obesity	-0.28	-0.89, 0.32	0.360	-0.16	-0.74, 0.43	0.559
Continuous obesity	0.42	-0.05, 0.90	0.082	0.36	-0.09, 0.82	0.115

ref= reference group; β = regression coefficient; CI = confidence intervals

Internalising and externalising symptoms ranged from 0 to 20.

Separate regression models were developed for each mental health outcome.

Model 1 controlled for baseline sex, race, family structure, caregiver education, caregiver employment, family income, pubertal status at age 11, and baseline BMI z-score at age 7.

Model 2: Model 1 with an additional adjustment for caregiver-reported baseline mental health at age 7 to assess changes in mental health from age 7 to 17.

Table S2. Associations between reversed (vs. continuous) obesity and self-reported follow-up mental health at age 17 (n= 975) *(see Table 3 in the main document for examining caregiver-reported follow-up mental health)*

**Obesity trajectories (baseline at age 7 and follow-up at age 17)** ***(ref = continuous obesity)***	**Model 1**			**Model 2**		
	**β**	**95% CI**	**p-value**	**β**	**95% CI**	**p-value**
**Dependent variable: Internalising symptoms**						
Reversed obesity	-0.82	-1.31, -0.32	0.001	-0.78	-1.26, -0.31	0.001
**Dependent variable: Externalising symptoms**						
Reversed obesity	-0.74	-1.31, -0.17	0.011	-0.55	-1.11, 0.01	0.052

ref= reference group; β = regression coefficient; CI = confidence intervals

Internalising and externalising symptoms ranged from 0 to 20.

Separate regression models were developed for each mental health outcome.

Model 1 controlled for baseline sex, race, family structure, caregiver education, caregiver employment, family income, pubertal status at age 11, and baseline BMI z-score at age 7.

Model 2: Model 1 with an additional adjustment for caregiver-reported baseline mental health at age 7 to assess changes in mental health from age 7 to 17.

Table S3. Associations between trajectory groups of obesity and mental health between ages 11 and 17 (n= 8,469)

**Obesity trajectories (baseline at age 11 and follow-up at age 17)** ***(ref = never developed obesity)***	**Model 1**			**Model 2**		
	**β**	**95% CI**	**p-value**	**β**	**95% CI**	**p-value**
**Dependent variable: Internalising symptoms**						
Developed obesity	1.04	0.66, 1.43	<0.001	0.73	0.40, 1.06	<0.001
Reversed obesity	0.43	-0.05, 0.92	0.080	0.25	-0.16, 0.66	0.235
Continuous obesity	1.53	0.98, 2.09	<0.001	0.86	0.46, 1.26	<0.001
**Dependent variable: Externalising symptoms**						
Developed obesity	0.61	0.25, 0.97	0.001	0.19	-0.12, 0.50	0.230
Reversed obesity	0.17	-0.39, 0.74	0.543	0.07	-0.39, 0.54	0.758
Continuous obesity	0.62	0.01, 1.24	0.048	0.07	-0.35, 0.50	0.731

β = regression coefficient; CI = confidence intervals

Internalising and externalising symptoms range from 0 to 20.

Separate regression models were developed for each mental health outcome.

Model 1 controlled for baseline sex, race, family structure, caregiver education, caregiver employment, family income, pubertal status at age 11, and baseline BMI z-score at age 11.

Model 2: Model 1 with an additional adjustment for baseline mental health at age 11 to assess changes in mental health from age 11 to 17.

Table S4. Associations between reversed (vs. continuous) obesity and mental health between ages 11 and 17 (n= 1,625)

**Obesity trajectories (baseline at age 11 and follow-up at age 17)** ***(ref = continuous obesity)***	**Model 1**			**Model 2**		
	**β**	**95% CI**	**p-value**	**β**	**95% CI**	**p-value**
**Dependent variable: Internalising symptoms**						
Reversed obesity	-0.78	-1.30, -0.25	0.004	-0.45	-0.91, 0.00	0.051
**Dependent variable: Externalising symptoms**						
Reversed obesity	-0.33	-0.91, 0.24	0.258	0.01	-0.45, 0.47	0.969

β = regression coefficient; CI = confidence intervals

Internalising and externalising symptoms range from 0 to 20.

Separate regression models were developed for each mental health outcome.

Model 1 controlled for baseline sex, race, family structure, caregiver education, caregiver employment, family income, pubertal status at age 11, and baseline BMI z-score at age 11.

Model 2: Model 1 with an additional adjustment for baseline mental health at age 11 to assess changes in mental health from age 11 to 17.

Table S5. Associations between the number of previous obesity episodes from ages 7 to 14 and current self-reported follow-up mental health in participants with and without current obesity at age 17 *(see Table 4 in the main document for examining caregiver-reported follow-up mental health)*

**Previous obesity episodes from ages 7 to 14**	**Participants with obesity at age 17 (n=1,479)**						**Participants without obesity at age 17 (n=5,767)**					
	**Model 1**			**Model 2**			**Model 1**			**Model 2**		
(ref = never developed obesity)	**β**	**95% CI**	**p-value**	**β**	**95% CI**	**p-value**	**β**	**95% CI**	**p-value**	**β**	**95% CI**	**p-value**
**Dependent variable: Internalising symptoms**												
Obesity at one time	-0.05	-0.70, 0.60	0.878	-0.19	-0.85, 0.47	0.578	0.12	-0.29, 0.52	0.578	0.05	0.35, 0.44	0.818
Obesity at two/three times	0.12	-0.40, 0.64	0.657	-0.04	-0.49, 0.56	0.888	0.29	-0.25, 0.83	0.292	0.28	0.26, 0.80	0.308
**Dependent variable: Externalising symptoms**												
Obesity at one time	-0.22	-0.82, 0.37	0.458	-0.28	-0.85, 0.30	0.344	0.03	-0.42, 0.48	0.898	0.01	-0.40, 0.42	0.969
Obesity at two/three times	-0.24	-0.73, 0.25	0.335	-0.23	-0.70, 0.25	0.347	0.28	-0.22, 0.78	0.272	0.29	-0.20, 0.79	0.245

ref= reference group; β = regression coefficient; CI = confidence intervals

Internalising and externalising symptoms ranged from 0 to 20.

Separate regression models were developed for each mental health outcome.

Model 1 controlled for current sex, race, family structure, caregiver education, caregiver employment, family income, and puberty status at age 11.

Model 2: Model 1 with an additional adjustment for caregiver-reported baseline mental health at age 7 to assess changes in mental health from age 7 to 17.

Table S6. Associations between the number of previous obesity episodes from ages 7 to 14 and current mental health at age 17 (n=7,246)

**Previous obesity episodes from ages 7 to 14** *(ref = never developed obesity)*	**Models without controlling for current obesity at age 17**						**Models controlling for current obesity at age 17**					
	**Model 1**			**Model 2**			**Model 1**			**Model 2**		
	**β**	**95% CI**	**p-value**	**β**	**95% CI**	**p-value**	**β**	**95% CI**	**p-value**	**β**	**95% CI**	**p-value**
**Dependent variable: Internalising symptoms**												
Obesity at one time	0.28	0.04, 0.59	0.082	-0.07	-0.22, 0.36	0.640	0.04	-0.28, 0.35	0.825	-0.15	-0.44, 0.15	0.322
Obesity at two/three times	0.43	0.16, 0.71	0.002	0.30	0.04, 0.57	0.024	-0.04	-0.38, 0.30	0.834	-0.12	-0.45, 0.20	0.458
**Dependent variable: Externalising symptoms**												
Obesity at one time	0.18	-0.13, 0.49	0.247	0.04	-0.23, 0.31	0.758	0.05	-0.28, 0.37	0.782	-0.02	-0.29, 0.26	0.894
Obesity at two/three times	0.06	-0.18, 0.29	0.624	-0.04	-0.25, 0.18	0.746	-0.21	-0.51, 0.10	0.180	-0.15	-0.42, 0.11	0.259

ref= reference group; β = regression coefficient; CI = confidence intervals

Internalising and externalising symptoms ranged from 0 to 20.

Separate regression models were developed for each mental health outcome.

Model 1 controlled for current sex, race, family structure, caregiver education, caregiver employment, family income, and puberty status at age 11.

Model 2: Model 1 with an additional adjustment for mental health at age 7 to assess changes in mental health from age 7 to 17.
